# Distinctive behaviour of live biopsy-derived carcinoma cells unveiled using coherence-controlled holographic microscopy

**DOI:** 10.1371/journal.pone.0183399

**Published:** 2017-08-28

**Authors:** Břetislav Gál, Miroslav Veselý, Jana Čolláková, Marta Nekulová, Veronika Jůzová, Radim Chmelík, Pavel Veselý

**Affiliations:** 1 St. Anne's University Hospital (FNUSA), Department of Otorhinolaryngology and Head and Neck Surgery, Brno, Czech Republic; 2 CEITEC—Central European Institute of Technology, Brno University of Technology, Brno, Czech Republic; 3 Institute of Physical Engineering (IPE), Faculty of Mechanical Engineering, Brno University of Technology, Brno, Czech Republic; 4 Regional Centre for Applied Molecular Oncology (RECAMO), Masaryk Memorial Cancer Institute, Brno, Czech Republic; Pennsylvania State Hershey College of Medicine, UNITED STATES

## Abstract

Head and neck squamous cell carcinoma is one of the most aggressive tumours and is typically diagnosed too late. Late diagnosis requires an urgent decision on an effective therapy. An individualized test of chemosensitivity should quickly indicate the suitability of chemotherapy and radiotherapy. No ex vivo chemosensitivity assessment developed thus far has become a part of general clinical practice. Therefore, we attempted to explore the new technique of coherence-controlled holographic microscopy to investigate the motility and growth of live cells from a head and neck squamous cell carcinoma biopsy. We expected to reveal behavioural patterns characteristic for malignant cells that can be used to imrove future predictive evaluation of chemotherapy. We managed to cultivate primary SACR2 carcinoma cells from head and neck squamous cell carcinoma biopsy verified through histopathology. The cells grew as a cohesive sheet of suspected carcinoma origin, and western blots showed positivity for the tumour marker p63 confirming cancerous origin. Unlike the roundish colonies of the established FaDu carcinoma cell line, the SACR2 cells formed irregularly shaped colonies, eliciting the impression of the collective invasion of carcinoma cells. Time-lapse recordings of the cohesive sheet activity revealed the rapid migration and high plasticity of these epithelial-like cells. Individual cells frequently abandoned the swiftly migrating crowd by moving aside and crawling faster. The increasing mass of fast migrating epithelial-like cells before and after mitosis confirmed the continuation of the cell cycle. In immunofluorescence, analogously shaped cells expressed the p63 tumour marker, considered proof of their origin from a carcinoma. These behavioural traits indicate the feasible identification of carcinoma cells in culture according to the proposed concept of the carcinoma cell dynamic phenotype. If further developed, this approach could later serve in a new functional online analysis of reactions of carcinoma cells to therapy. Such efforts conform to current trends in precision medicine.

## Introduction

Cancer therapy is currently progressing towards the individualization of treatment guided by evidence based on individual tumour properties [1]. Live cancer cells propagated in vitro from biopsy have exemplified a plausible source of information for assessing solid tumour sensibility to therapy. In addition, the attributes of these cells should also provide a contribution to the prognosis [2]. Many chemosensitivity assays have been developed. Most of these assays rely on an evaluation of the extent of cell death caused by the presence of an anticancer drug [3]. However, none of these methods have become part of clinical practice. In 2004, an American Society of Clinical Oncology panel did not find sufficient evidence to support the routine use of in vitro anticancer drug resistance tests and advocated the inclusion of these potentially important research methods in prospective clinical trials. Since 2004, this situation has not changed. There is no regular chemosensitivity or chemoresistance assay save for ovarian carcinoma, which, based on current evidence, would be sufficient to support usage in oncology practice [4].

The in vitro motility of tumour cells is associated with the local invasiveness and metastatic potential of experimental tumours in vivo [5,6]. Recently, Zhao et al. [7] also provided evidence for salivary adenoid cystic carcinoma, reporting that the down-regulation of the microtubule-associated tumour suppressor gene (MTUS1) expression contributes to the proliferation, migration and invasion abilities of this tumour as assayed in vitro. There are numerous methods to evaluate cell motility in vitro under varying conditions. Nevertheless, the application of these methods is limited, and their clinical impact remains minimal. However, the qualified exploitation of the understanding of the regulation of migration and model invasiveness in vitro for the examination of individual ex vivo cultured carcinoma cells remains crucial for progress in cancer diagnostics and therapy.

Our development of coherence-controlled holographic microscopy (CCHM) has offered us an opportunity to examine the reactions of live cells. CCHM quantitative phase imaging (QPI), which can be feasibly exploited even through turbid media [8] to measure tiny differences in cell motion [9], presents an innovative objective analysis of live cancer cells in vitro achieved by simultaneously imaging the cell shape and position and measuring changes in the cell mass, i.e., growth. Indeed, CCHM in a Q-PHASE microscope (Tescan Orsay Holding, a.s., Brno, Czech Republic) recently contributed to the differentiation of oncosis from apoptosis [10] and the recognition of entosis, which emerged as a particularly important type of ostensible cell death but is potentially required for tumour survival [11].Head and neck squamous cell carcinoma (HNSCC) is a pressing clinical problem. The available treatments based on a multimodal approach comprise surgery, chemotherapy, radiotherapy and biological therapy while emphasizing organ preservation. HNSCC has been selected in the present study to examine the promise of the CCHM method to contribute to clinical practice by the in vitro identification of live carcinoma cells. If accomplished, these cells from individual tumour could subsequently be used in the screening for the best therapy option.

We acquired primary carcinoma cells from an HNSCC biopsy and employed molecular biology to confirm their cancerous origin. Subsequently, we used CCHM QPI to recognize behavioural traits. These efforts should further the current understanding of the in vitro identification of individual carcinoma live cells.

## Materials and methods

### Patient selection

We used three HNSCC tumours obtained from patients selected according to the inclusion and exclusion criteria for the investigation of tumour biopsies in the present study, which was approved by the FNUSA Ethical Commission.

Inclusion criteria: biopsy proven; operable and inoperable; measurable stage III, IVA, IVB squamous-cell carcinoma of the oral cavity, oropharynx, hypopharynx or larynx with no previous malignancy; age between 18 and 75 years; good performance status (WHO grade 0 – 1); adequate laboratory tests (blood count and creatinine clearance); no symptomatic altered hearing; no peripheral neuropathy grade ≥ 2; and informed consent.

Exclusion criteria: generalized disease and refusal to the patient to participate in the study.

### Tumour biopsy

We obtained, within the framework of the diagnostic protocol, tumour samples using surgical excision from the central part of the solid tumour tissue. We divided the volume of the approximately 500-mm^3^ sample into halves. One half was used for regular histopathology examination. The other half was promptly transported in cold culture medium to the Cell Biology Laboratory of the Experimental Biophotonics (CEITEC).

### Cell culture

For the primary culture from biopsy, specimen cells were enzymatically released through digestion in 0.05% collagenase A (Roche, Penzberg, Germany) or 0.25% trypsin (Sigma-Aldrich, Prague, Czech Republic). We also cultivated cells outgrowing from tumour tissue fragments. Plain TC plastic or glass-bottomed dishes were used as culture vessels. EMA medium (a kind gift from Dr. Eva Matoušková) was H-MEM supplemented with non-essential amino acids (Sigma-Aldrich, Prague, Czech Republic), 1 mM sodium pyruvate, 0.3 g/L L-glutamine, 0.5 μg/ml hydrocortisone, 5 μg/ml insulin, 10^−10^ M cholera toxin, 5 ng/ml epidermal growth factor (all these chemicals from Sigma-Aldrich, Prague, Czech Republic), 10% calf serum (ZVOS Hustopece, Czech Republic) and 2% foetal bovine serum (Sigma-Aldrich, Prague, Czech Republic), penicillin, and streptomycin [12]. The cells were fed either plain EMA medium or EMA medium supplemented with 20% MEM and 10% calf serum conditioned on semiconfluent NIH 3T3 cells for one day and subsequently centrifuged and filtered. For time-lapse microscopy, the EMA medium was modified as follows: phenol red was omitted, sodium bicarbonate concentration was lowered to one-third (0.3 g/L) and 20 mM of non-volatile buffer TES (Sigma-Aldrich, Prague, Czech Republic) was added, and the pH set to 7.4. We subsequently used the same media in the continuing cultivation of the isolated cells.

FaDu cell line (ATCC HTB-43) was established from a punch biopsy of a human hypopharyngeal tumour [13]. It is one of the most widely used HNSCC cell models and it is also a part of ATCC Head and Neck Cancer Panel (ATCC TCP-1012). FaDu has been subjected to genomic analysis and pharmacological profiling [14,15] and has already been used as a control for newly established HNSCC cell line [16]. The established FaDu HNSCC cell line was maintained in standard culture medium (H-MEM with 5% foetal bovine serum).

### Light microscopy

■ Nikon Eclipse TS100-F equipped with Zernike phase contrast LWD objectives for the regular assessment of the cell culture and static photography and short time-lapse recording of ongoing cell morphological changes.
■ Olympus Bx41 with Olympus XM10 camera for fluorescence imaging.
■ NIKON confocal A1R for DIC and fluorescence imaging.
■ Q-PHASE multimodal holographic microscope (Tescan Orsay Holding, a.s., Brno, Czech Republic) for the investigation of complex cell behaviour using the CCHM QPI method.

### SDS-PAGE and western blot

The cells were trypsinized, washed in PBS and resuspended in lysis buffer (150 mM NaCl, 1% Nonidet P-40, 50 mM Tris-HCl, pH 8.0, 5 mM EDTA, pH 8.0, and protease inhibitor cocktail). The protein concentration was measured using a Bradford protein assay, and 20 μg of total protein in the NuPAGE LDS Sample Buffer (Life Technologies, USA) was loaded onto 10% polyacrylamide gels, separated and transferred onto nitrocellulose membranes using the Mini-PROTEAN Electrophoresis System (Bio-Rad, USA) for 90 min, applying 100 V in the transfer buffer (240 mM Tris, 190 mM glycine, and 20% methanol). The membranes were blocked in 5% non-fat milk in PBS with 0.1% Tween and incubated overnight with primary antibodies at 4°C. Primary antibodies used: ΔNp63-1.1 (ΔNp63, Moravian Biotechnology, Czech Republic), DC-10 (cytokeratin 18, Exbio, Prague, Czech Republic), DO-1 (p53, Moravian Biotechnology, Czech Republic), NCL-L-EGFR (EGFR, Novocastra, UK), vimentin—Dako—Clone V9, E-cadherin—Cell Signaling - 24E10, N-cadherin—Cell Signaling—D4R1H, AC40 (actin, Sigma-Aldrich, USA). The membranes were subsequently incubated with HRP-conjugated secondary antibody (RAM-Px, Dako, Denmark) for 1 h at room temperature. The signal was detected using ECL reagent (Amersham Pharmacia Biotech, UK).

### Immunofluorescence

The cells were grown on coverslips for 24 h, washed in phosphate-buffered saline (PBS) and fixed in 4% formaldehyde in PBS for 20 min at room temperature. After washing in PBS, the cells were permeabilized in 0.1% Triton X-100 in PBS for 5 min. To block the nonspecific binding of the antibodies, cells were incubated with 3% bovine serum albumin (BSA) in PBS for 20 min. The cells were incubated with diluted antibody in 3% BSA in PBS for 1 h at room temperature. After 3 x 5 min washing with PBS, the cells were incubated with secondary antibody in 3% BSA in PBS for 1 h at room temperature in the dark. After 3 x 5 min wash with PBS, the cell nuclei were stained with 1 μg/ml Hoechst for 5 min. The coverslips were mounted with Vectashield H-1000 mounting medium (Vector Laboratories, USA) and stored in the dark at 4°C. Primary and secondary antibodies used: ΔNp63-1.1 (ΔNp63, Moravian Biotechnology, Czech Republic), SFI-6 (p63, DCS-Innovative Diagnostik-Systeme, Germany), MOC-31 (EpCAM, Abcam, UK), and goat anti-mouse IgG DyLight-488 conjugate (Thermo Fisher Scientific, USA).

### Coherence-controlled holographic microscopy (CCHM)

The first diffraction order of the diffraction grating is separated in the reference arm to provide a tilted off-axis reference beam and to ensure achromaticity and spatial invariance of the interferometer. Such a configuration equips CCHM QPI with the benefits of both spatially and temporally incoherent illumination. CCHM incoherent light imaging improves the image quality of QPI compared to coherent-illumination digital holographic microscopy (DHM) [17] by suppressing coherence noise (speckles), interferences and diffraction artefacts, while the lateral resolution is enhanced closer to standard light [17, 18]. The complete CCHM set-up brings about the halo artefact suppression and an advantage of a single shot hologram registration with the CCD camera [17, 19]. All the factors mentioned contribute to the fast and sensitive detection of cell outlines that enables reliable segmentation of cells making CCHM particularly suitable for study of living cells dynamics.

To accommodate cells in Q-PHASE, we used various homemade or commercial (IBIDI, Germany) observation chambers.

#### Image data processing

The Q-PHASE microscope produces holograms captured using a CCD camera (Ximea MR4021MC-BH). Q-PHASE software provides the online reconstruction of phase shifts in radians into dry mass density (pg/µm^2^) [18, 20, 21]. The error range of mass measurement was calculated on fixed cells under the same conditions and was approximately 1% of the total cell mass.

#### Cell cycle time estimation

Qualified estimation of the cell cycle time without the need of measuring the whole cell cycle can be achieved by calculating the cell mass doubling time from measuring the relative growth rate [22, 23]. Measurements of the cell mass increase lasting for at least 3 hours are needed. Non-applicable situations, such as cells colliding with debris, small fragments attached to the substratum, cells leaving the field of view or cells in mitosis, must be excluded from data processing. In addition, situations in which the segmentation algorithm fails while the cells crawl over or are in tight contact with each other are also excluded. Despite the excluded data points, we could reliably fit the data and calculate the time that a cell potentially requires to double its mass and presumably complete the cell cycle.

#### Cell migration

Migration trajectory and speed of migration for statistical analysis were derived from localization of the mass centroid and its translocation. We measured the translocation of the centre of the cell mass over 3 hours at five-minute interval between frames for the centroid stabilization in order to avoid the error of accumulating small translocations produced through cellular in situ motility. We defined the migration trajectory as the sum of the Euclidean distances between two following time points and actual speed from the length of the partial trajectory.

### Statistics

Student’s un-paired t-test was done using Matlab, MathWorks, Inc. and boxplot evaluation was done using OriginPro 2016, OriginLab, Corp.

## Results

### Primary cultures from individual HNSCC

SACR1 tumour: Advanced squamous cell carcinoma of the hypopharynx, T3N2bM0, intermediate grade (G2), p53 neg. Patient: male, 64 years of age. SACR1 cells were harvested using collagenase or trypsin digestion of the biopsy fragments. Within one week, some epithelial-like cells, which mesenchymal-like cells later overgrew, were observed.

SACR2 tumour: Squamous cell carcinoma of the oral cavity, T2N0M0, intermediate grade (G2), p53 neg. Patient: male, 73 years of age.

**Fig 1:**
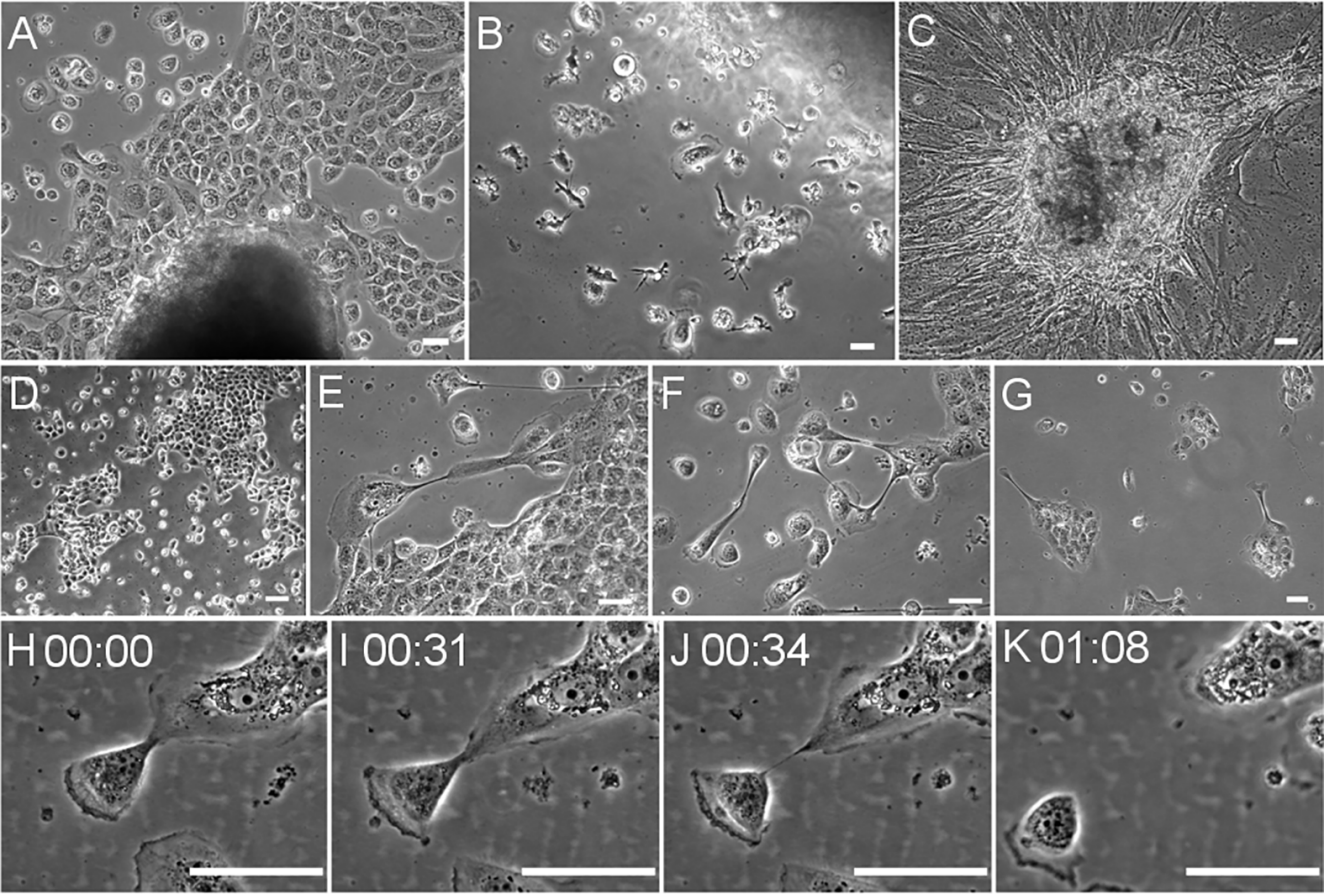
SACR2 cells early in culture. (A) Ex vivo fragment with outgrowing epithelial-like sheet of cells; (B) Ex vivo fragment with individual cells dispersed around the fragment and mixed with tumour-associated ramified macrophages that subsequently vanish. (C) Ex vivo fragment of healthy tissue from the vicinity of an SACR2 tumour with outgrowing mesenchymal cells. (D) Irregular shape of primary colonies. (E) Pleomorphic cell phenotype in parts of a semi-confluent culture. (F) Variability of cell shapes in sparse culture. (G) Unusual colony shapes invoking the impression of latent propensity to invasion. (H-K) active release within one hour of an SACR2 cell from a projection of the colony at lab temperature. Scale bars, 50 µm.

SACR2 cells were obtained after trypsinization and fragment culture. The primary cells emerging from the tumour fragments gave rise to the only growing epithelial-like cell population obtained (Fig 1A). Surprisingly, there were no fibroblast-like cells competing with the epithelial-like cells, which were subsequently confirmed to originate from the carcinoma.

SACR3 tumour: Advanced squamous cell carcinoma of the oropharynx, T3N2aM0, high grade (G3), p53 neg. Patient: male, 57 years of age.

SACR3 cells were derived from fragment cultures only. Reflecting problems with the insufficient attachment of fragments to the culture substratum, we harvested only some cells with peculiar non-epithelial morphotypes.

#### Stationary characterization of live SACR2 cells

**Fig 2:**
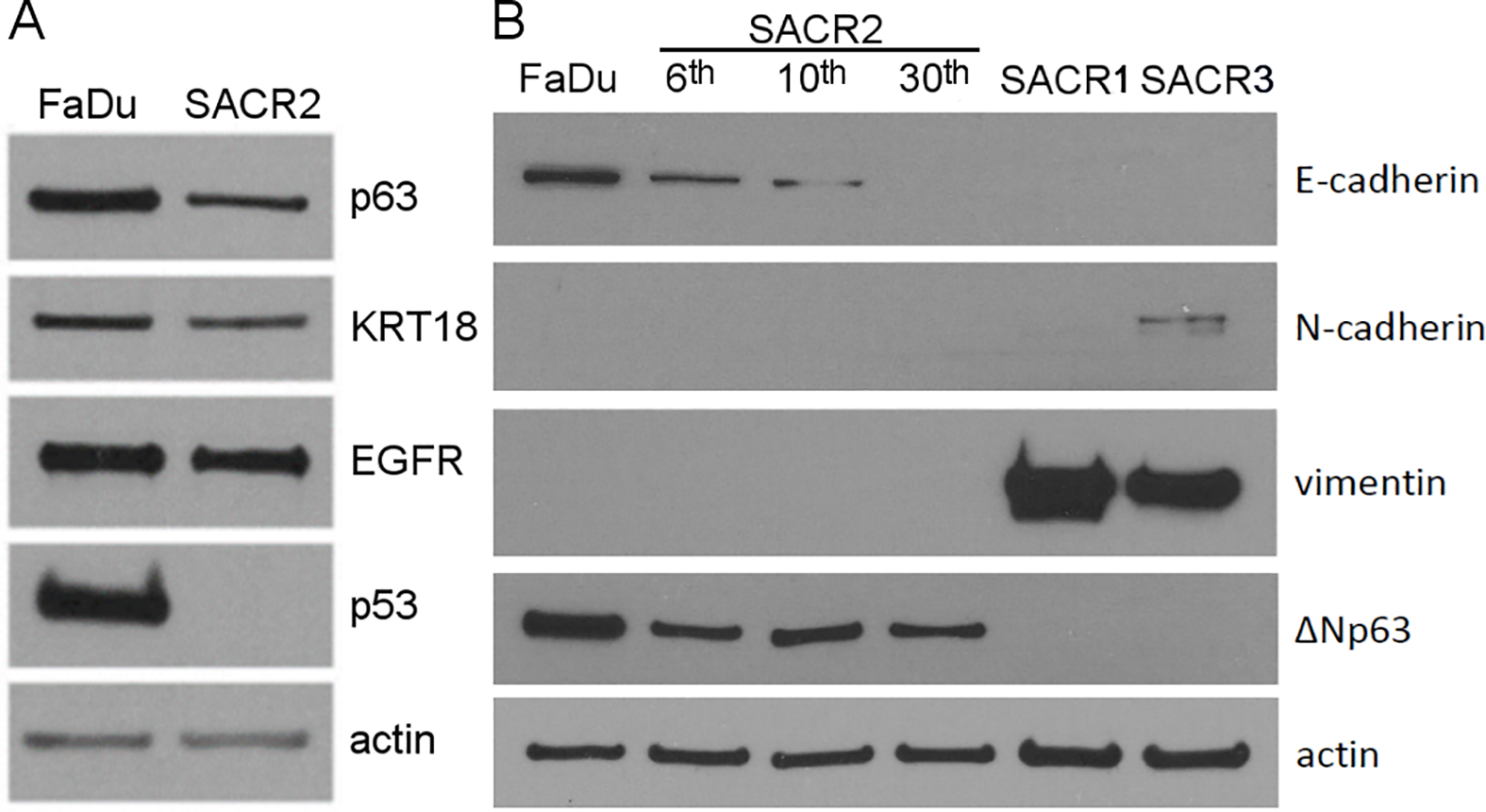
Western blots for tumour markers. (A) Comparison of FaDu with SACR2 cells shows high similarity in p63, KRT18 and EGRF expression and an absence of p53 in SACR2 cells. (B) SACR2 cells compared with FaDu cells show decreasing expression of E-cadherin with increasing passages (6^th^, 10^th^, 30^th^) and almost the same expression of ΔNp63. Vimentin was expressed only in SACR1 and 3 cells, and N-cadherin was only slightly expressed in SACR3 cells.

**Fig 3:**
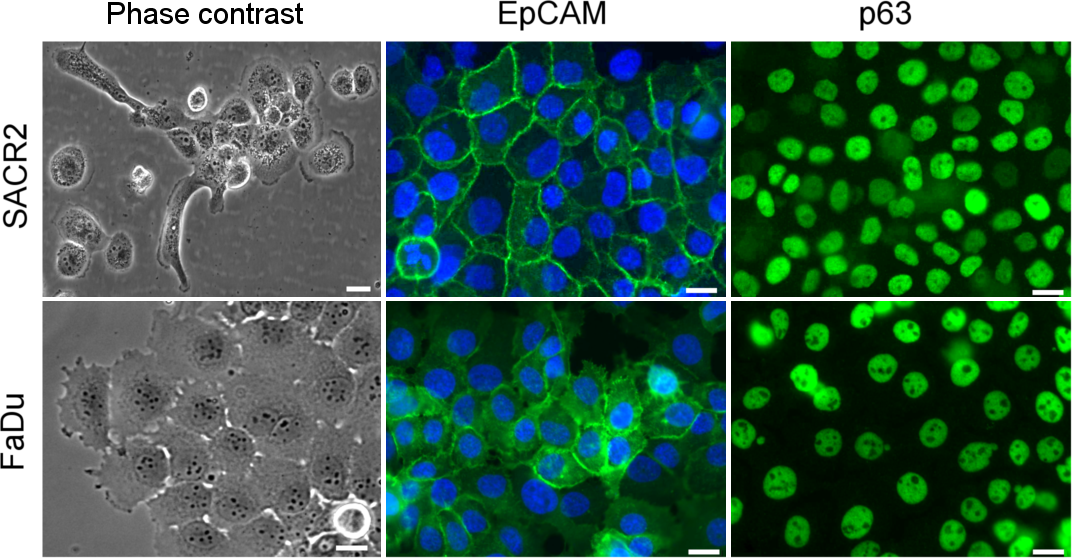
Comparison between SACR2 epithelial cells (10^th^ passage) and the FaDu HNSCC cell line. Phase contrast: SACR2 cells still show an irregular shape, both in terms of cells and colonies, while polygonal FaDu cells form regular rounded colonies. EpCAM and p63 are expressed in the individual cells of both populations. Scale bars, 20 µM.

We selected SACR2 cells because of the early epithelial-like morphology (Fig 1A) for further study and comparison with the established HNSCC cell line FaDu [13]. SACR2 cells in culture showed an epithelial-like shape, and on the population level, they expressed the p63 tumour marker of squamous cell carcinoma (Fig 2A) [24, 25]. SACR2 cells in static observation of the dense layer appeared as tightly packed ordinary epithelial cells (Fig 1A, 1D and 1E). However, the morphology of the growing colonies was irregularly shaped and jagged (Fig 1D and 1G), which differed from the ordinary roundish colonies of the HNSCC FaDu cells (Fig 3). The image of an actively migrating isolated cell protruding into the free culture space followed by other cells provided a strong impression of the collective invasion of carcinoma cells (Fig 1D and 1G, Fig 3). This notion confirmed the short time-lapse observation that despite being taken only at lab temperature, the active release of the leading cell from the colony projection (Fig 1H–1K) was observed within one hour.

#### Western blots for tumour markers

SACR2, similar to FaDu cells, expressed p63, KT18, EGFR, ΔNp63 and decreased expression of E-cadherin but not p53 (Fig 2A). SACR1 and SACR3 expressed vimentin, which was not detected in FaDu or SACR2 cells. N-cadherin was only marginally detected in SACR3 cells (Fig 2B). We considered confirmation of the p63 expression in the SACR2 cells as evidence of carcinoma origin [24, 25].

#### Dynamic CCHM QPI analysis of SACR2 cells

**Fig 4:**
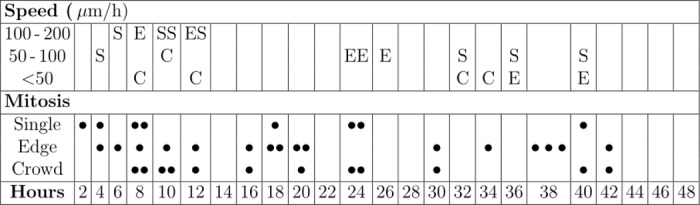
Overview of SACR2 cell activities manifested over 48 hours of time-lapse recording at the 9^th^ passage (Q-PHASE, 10x objective lens, field of view 376 x 376 μm^2^, 8-sec intervals). Mitoses and the measurement of short lasting maximum migration speed were recorded in relationship to the “social” situation of the cell. From all 36 mitoses, 8 events were observed in single migrating cells (S), 16 events were observed in cells at the edge of the moving group (E), and 12 events were observed inside the group (C).

For CCHM, we investigated the SACR2 cells for dynamic morphology, motility as the ruffling of the leading cell periphery engaged in migration and in cell-to-cell collisions. We measured these characteristics as related to cell mass in two stages of SACR2 cells continuously cultured in vitro. The first stage was in the fourth passage (55 days after seeding), when cells other than epithelial morphotypes could still likely occur, e.g., fibroblasts and macrophages, as in primary culture (Fig 1B and 1C). The second stage was in the ninth passage (110 days after seeding), and 48-hour time-lapse recording provided substantial information on the nature of the SACR2 cells. Subsequently, when defrosted from cells frozen after the sixth passage (after 120 days in culture, of which 53 days were spent frozen in liquid nitrogen), SACR2 cells were examined for the stability of the dynamic phenotype. In all instances, the patterns of SACR2 cell motility, migration and growth, also indicated by mitotic activity, were similar. Fig 4 reviews SACR2 events of maximum migration speed and mitotic activity over a period of 48 hours of time-lapse recording at 8-sec intervals between frames. Within the field of view, we observed approximately one hundred cells migrating individually or in a group there. Cell activity was evaluated in relationship to the “social” situation of the cell. The maximum speed either achieved by cells, either single or at the edge and leaving the moving crowd, reached almost 200 μm/h, which is approximately three times the speed of the group. This phenomenon indicates a type of random activation of migration activity in a part of the cell population. The observed cells produced 36 mitoses peaking between the 8th and 11th hour after onset. The duration of cell division varied between 20 and 100 minutes. Precise measurement was complicated by a delay in the final separation of the daughter cells, which frequently remained connected after telophase through a thin cable for some time during early G1. If mitoses were observed in subsequent quarters throughout, then declining numbers were detected: 14, 9, 6, and 7. Such a declining trend in mitotic activity and speed of migration apparently reflects a worsening of the nutritional conditions.

**Fig 5:**
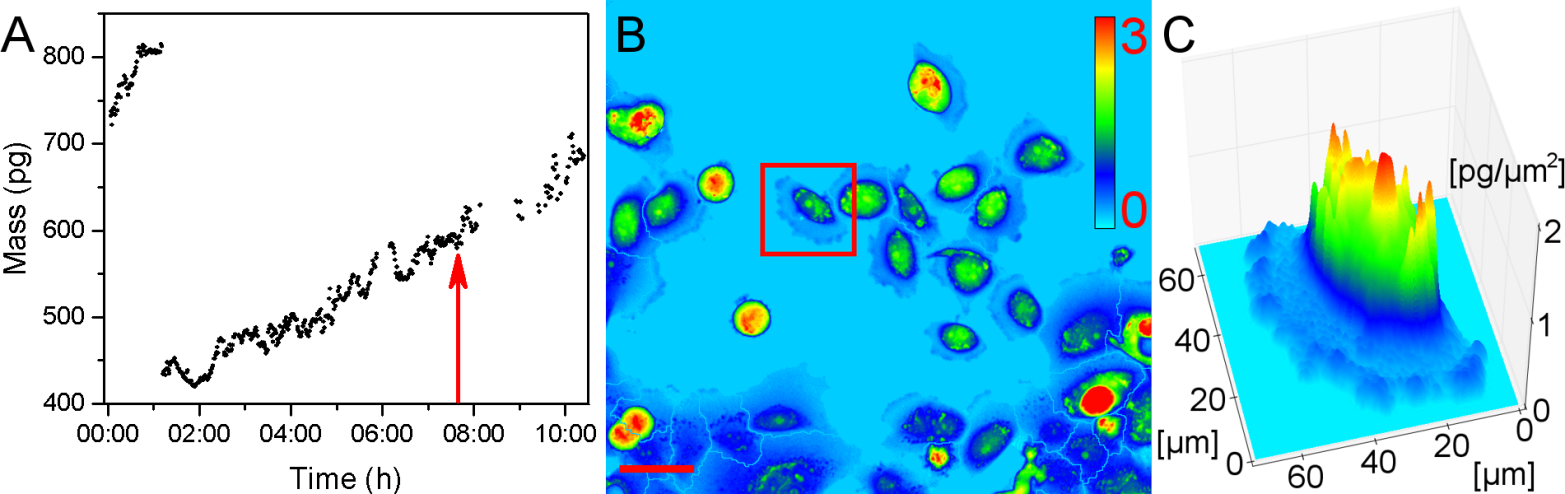
SACR2 cells at the 9^th^ passage. (A) Cell cycle measurement of a quickly migrating and dividing single cell. The steady gain in mass of the mother and one daughter cell indicates continuous cell cycling (doubling time 13.7 hours). The red arrow shows the daughter cell in the cell cycle, captured in the red square in the (B) pseudo-coloured quantitative phase image of the field of view. (C) The same cell is presented in quasi-3D of dry mass density, suggesting increased cell height. Calibration bar is in pg/μm^2^. Scale bar, 50 μm.

SACR2 cells had a highly plastic shape with a notable mass profile, suggesting a raised cell height (Fig 5C). They showed a ruffling periphery, even at 1-sec time lapse intervals, engaged in rapid migration (Fig 5B). Within the field of view, cells were migrating individually or in a group. Single moving cells repeatedly joined the group or actively broke the connection with the group and accelerated away individually and repeatedly, often undergoing mitosis along the way. When we measured the mass of such cells before and after mitosis, the continuing gain in mass indicated growth and continuous cell cycling (Fig 5A). Homotypic collisions between migrating cells led to a temporal cessation of undulation of both membranes, but only in the area in contact between these cells. There were no signs of cells engaged in underlapping or overlapping among the various cells examined.

#### Immunofluorescence for tumour markers

**Fig 6:**
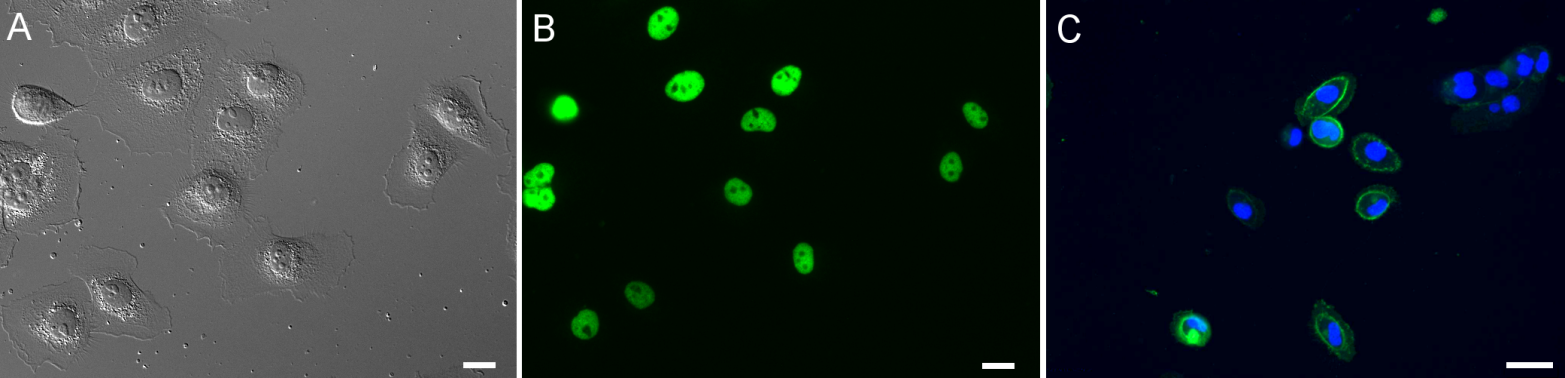
SACR2 cells in sparse culture. (A) DIC image shows migrating individual cells. (B) The same cells are p63 positive. (C) Peripheral EpCAM positivity of similar individual SACR2 cells (6^th^ passage, thawed) surroung cell nucleus (DAPI) confirm the difference of individual SACR2 epithelial cells from macrophages. Scale bars, 20 μm.

Epithelial-like morphotypes of SACR2 cells were investigated for tumour markers using fluorescence microscopy. The individual apparently migrating cells that in static picture of fixed cells showed ruffling of the cell leading periphery were also positive for p63 (Figs 3 and 6). In particular, EpCAM membrane staining (Fig 6C) in this type of cells also distinguished carcinoma cells from tumour-associated macrophages [24].

#### Comparison of SACR2 with FaDu cells

**Fig 7:**
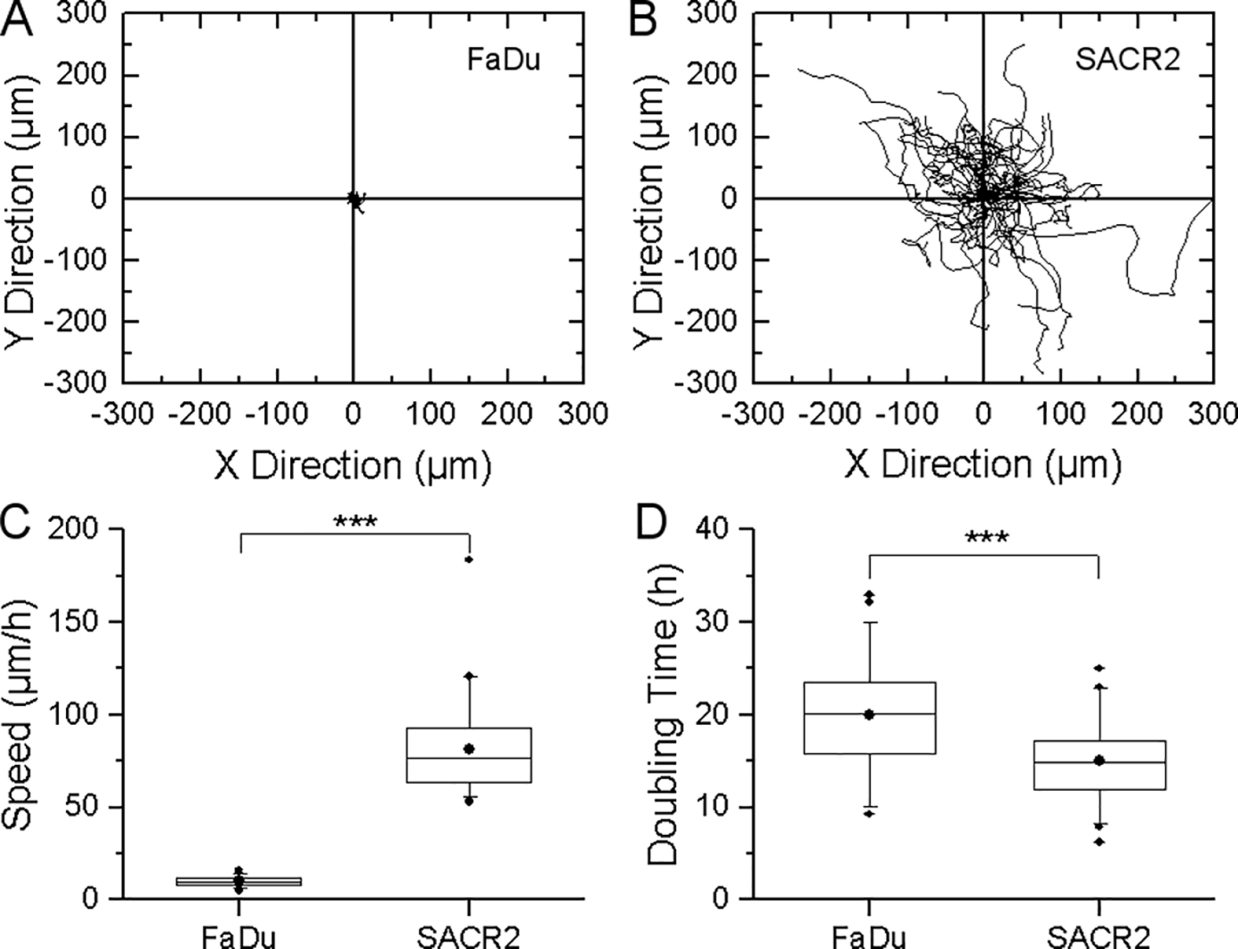
Comparison of SACR2 cells in the 9^th^ passage with the FaDu cell line. (A, B) Migration trajectories were defined as the sum of partial Euclidean distance measured for the translocation of the centre of the cell mass. 50 cells over 3 hours sampled at 5-minute interval. (C) The boxplot compares migration speed, which is significantly higher for SACR2 than FaDu cells. Data from A and B. (D) Doubling time was calculated from the growth rate during the available time period for estimation of probable cell cycle time. The boxplot indicates the shorter cell cycle time of SACR2 cells and thus apparently higher growth rate compared with FaDu cells. 45 cells sampled for 3hr at 5-minute interval for each culture. The dots in the boxes indicate the mean, the centreline is the median, the top of the box is the 25^th^ percentile, and the bottom of the box is the 75^th^ percentile. The whiskers indicate the 90^th^ percentiles (5^th^ and 95^th^), and the outliers are plotted (OriginPro 2016, OriginLab, Corp.). These differences between SACR2 and FaDu cell populations were statistically significant; *** denotes P values <0.0005.

A comparison of SACR2 cells selected from 48 hours lasting follow up in the ninth passage with the FaDu cells time-lapsed for 66 hours at 1 minute interval is shown in Fig 7. When tracked, the length of the trajectories were near zero for FaDu cells (Fig 7A), while that for SACR2 cells were up to 300 μm (Fig 7B). In Fig 7C, the boxplot representation confirms a significantly higher average speed of migration of SACR2 (in the 9th passage) compared with FaDu cells (50 cells from each time-lapsed record). The periods of highest speeds of SACR2 cells registered in Fig 4 lasted less than 3 hours and therefore these cells cannot be considered here. Doubling time was calculated from the exponential growth as the period of time that an individual cell potentially needs to double its mass and presumably accomplish the cell cycle through mitosis (Fig 7D). The boxplot indicates the shorter cell cycle time of SACR2 cells and thus apparently higher growth rate compared with FaDu cells (45 cells from each time-lapsed record).

## Discussion

We have used the technology of CCHM QPI for the evaluation of dynamic cell morphology and migration simultaneously with the growth of ex vivo cultured SACR2 head and neck squamous cell carcinoma cell population positive for p63.

SACR 1 and 3 had to be omitted for absence of p63 expression while expressing mesenchymal cell marker vimentin (Fig 2).

The patterns of cell behaviour verified through tumour markers detected using immunocytochemistry should eventually lead to the in vitro online identification of live carcinoma cells.

For SACR2 cells in static pictures, we first obtained an impression of the epithelial sheet of cohesive cells (Fig 1A), which were similar to those of the FaDu cell line (Fig 3). The first remarkable difference was the morphology of colonies with an invasion prone aspect (Fig 1D and 1G, Fig 3) that could initiate metastasis. Western blots subsequently revealed the expression of the tumour marker p63 [25, 26] in SACR2 cells, similar to FaDu cells (Fig 2).

This impression changed when high motility and migratory activity were observed in time-lapse recordings associated with mitoses and steadily increasing cell mass during migration. Data describing the dynamics of SACR2 cell behaviour suggested that these events might represent an ongoing process related or identical to the epithelial-mesenchymal transition, indicated by the intermediate phenotypes of the cells in transition [27]. We observed the loss of some epithelial markers, such as decreasing E-cadherin, ΔNp63 and KT18, from SACR2 cells, while not yet acquiring the mesenchymal marker vimentin (Figs 2, 3 and 6).

Nevertheless, instead of showing only the onset of migration expected around the edge of the sheet of growing epithelial cells, we observed approximately one hundred growing and dividing cells that moved as a group and individually back and forth. Single cells frequently crawled for a short time at a rapid speed of almost up to 200 μm/h in a sideways manner, which is typical for fast migrating epithelial-like fish keratocytes [28]. The increased mass density of these cells (Fig 5B) signified an elevated cell body, considered a marker of neoplastic cells [29, 30]. The average speed was lower (Fig 7C) because of the 3-hour tracking of cells and measuring of growth for the cell mass doubling time calculation (Fig 7D), indicating a shorter cell cycle time for SACR2 cells than for FaDu cells (Fig 7D). This finding highlights the value of using CCHM QPI observation of primary cultures from biopsy ex vivo as soon as possible because it can accelerate the timely recognition of the true character of the observed cell population. However, the absence of migration in FaDu cells is likely related to the expression of epithelial markers. FaDu cells express p63 and p53, which is missing in SACR2 cells and in the original tumour. In FaDu cells, there is more ΔNp63, E-cadherin and KT18. Th conceivable confusion of an SACR2 cell with macrophages is eliminated not only by the regularity of cell divisions but also by EpCAM expression on the surface (Fig 6C) of an apparently migrating cell. This could also indicate that the oncogenic cleavage of EpCAM during the epithelial-mesenchymal transition [31] does not necessarily precede the onset of migration.

We suppose that capturing these patterns of cell behaviour was facilitated by the specific conditions of the fragment culture, which offered a suitable attachment option to support emerging mesenchymal traits in transitional cells. This observation could be considered a neglected advantage of the decreasingly common 2D cell culture style. Fragment culture also has the potential to uncover the heterogeneity of cells from an individual tumour and consider their interactions. However, the conditions for primary tumour fragment culture still need significant improvement.

Using QPI, we observed and measured the suspected progeny of epithelial-like cells of carcinoma cells growing out from tumour tissue fragments. The cells exhibited mitoses, growth and a high speed of migration. Using immunofluorescence, we detected tumour markers expressed in individual analogous cell types. In addition to the epithelial and epithelial-mesenchymal transition markers, the expression of p63 appears to be the main evidence for the neoplastic, and particularly HNSCC, origin of SACR2 [25, 32, 33, 34, 35, 36].

These findings suggested that CCHM QPI observation and measurement of carcinoma biopsy cells in action revealed the following cancerous traits characterizing the proposed carcinoma cell dynamic phenotype:

Highly changeable epithelial shape; elevated cell body; leading lamella rapid undulation; rapid sideways crawling; fast migrating groups instead of a concentrically growing cell colony or sheet; individually acting cells moving even faster than the group and heading in a different direction while gaining mass and going through mitosis in cycles of separating from the group and merging with it later; and preservation of the homotypic contact inhibition of locomotion.

All these traits of cell behaviour are amenable to change upon treatment with drugs. Consequently, not only cell survival or death can be evaluated. This scenario deepens quality of testing live cancer cells.

## Conclusions

We understand that the results are first steps to the timely online identification of the carcinoma cell dynamic phenotype achieved with the CCHM QPI method. The method in the next step, will have to be validated by testing cells from malignant and benign tumours. Correspondingly, the identification of cancerous cells through direct microscopic evaluation could subsequently serve an endeavour to devise e.g. an early chemosensitivity test of individual tumours or to validate migrastatics [37].

The recognition of cancerous cells from the dynamics of behavioural activity could bridge the gap between wide genomic knowledge versus uncertainty on the resulting phenotype of cancer cells [38]. Thus, this new understanding should promote progress in the envisaged applications of precision medicine.

## Supporting information

S1 Table Migration trajectories of individual FaDu cells (Fig 7A). Coordinates (x,y) in μm of cell center of mass taken at 5 minute interval.
(XLSX)

S2 Table Migration trajectories of individual SACR2 cells (Fig 7B). Coordinates (x,y) in μm of cell center of mass taken at 5 minute interval.
(XLSX)

S3 Table Migration speed of FaDu and SACR2 cells (Fig 7C). Boxplot metadata. (XLSX)

S4 Table Doubling time of FaDu and SACR2 cells (Fig 7D). Boxplot metadata. (XLSX)

S1 Video Behaviour of SACR2 cells in vitro. (MP4)

S2 Video Behaviour of FaDu cells in vitro. (MP4)
